# Supplementary material for: Structures of the T cell potassium channel Kv1.3 with immunoglobulin modulators
Source: Nat Commun. 2022 Jul 4;13:3854. doi: 10.1038/s41467-022-31285-5 (PMC9253088; doi:10.1038/s41467-022-31285-5)
Supplement: Supplementary file 1 — Supplementary Information [file 41467_2022_31285_MOESM1_ESM.pdf]

SUPPLEMENTARY FIGURES AND TABLES

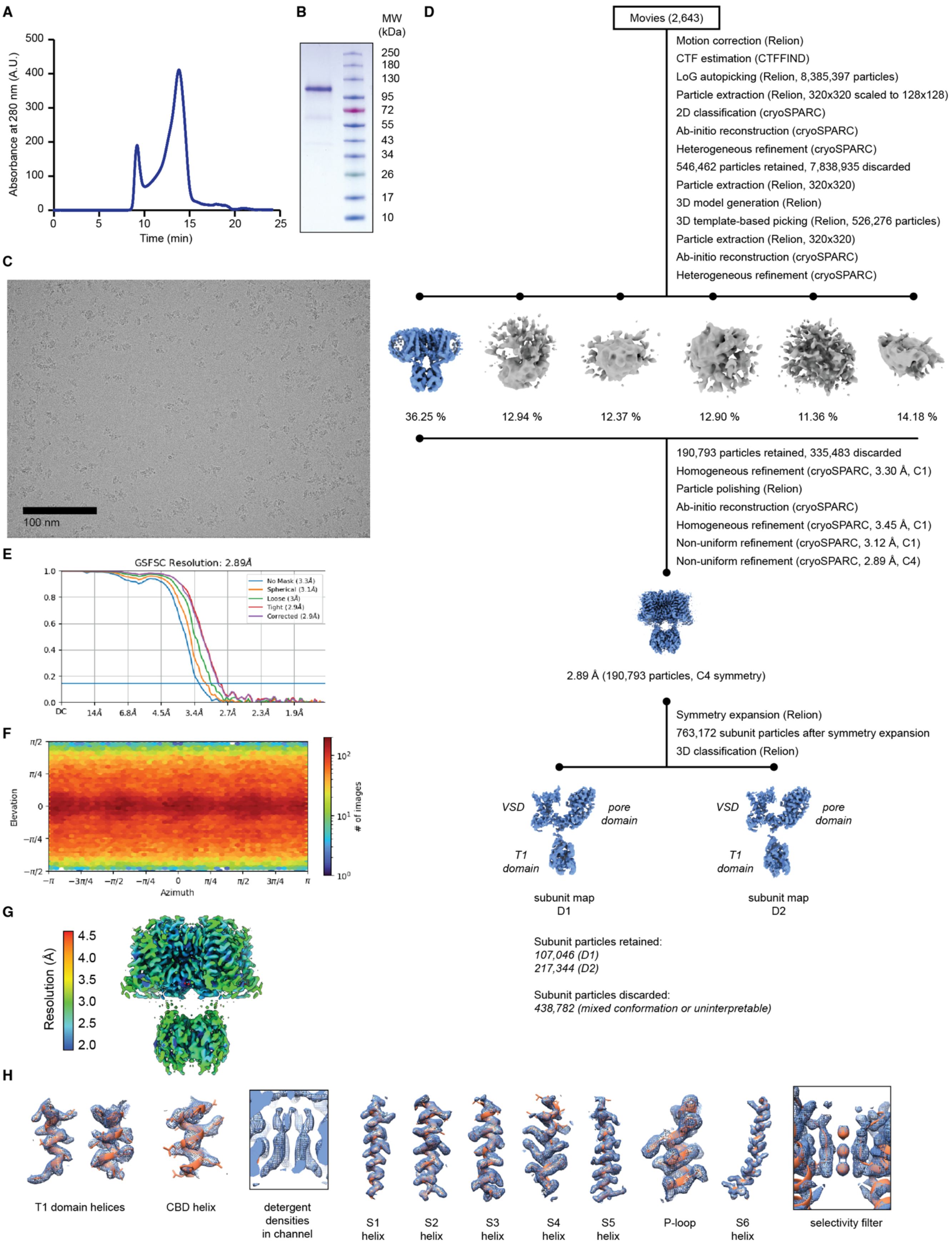

Supplementary Figure 1. Purification, cryo-EM, and structure determination for human Kv1.3. (A-B) Gel filtration trace (A) and SDS-PAGE (B) for purified human Kv1.3. This purification was independently repeated three times with similar results. **(C)** Representative cryo-EM image of Kv1.3. Imaging was independently repeated four times with similar results. **(D)** Data processing workflow for Kv1.3. Cryo-EM density maps are color-coded as blue or gray according to whether they are retained or discarded, respectively. The percentage of particles in each class from heterogeneous refinement is given. The final two maps are for individual Kv1.3 subunits in the D1 (left) and D2 (right) conformations isolated by symmetry expansion and 3D classification. **(E)** Fourier shell correlation (FSC) curves as generated by cryoSPARC. **(F)** Angular distribution plot for particles in the reconstruction as generated by cryoSPARC. **(G)** Local resolution heat map. Data was processed with C1 and C4 symmetry as indicated, with the final map refined with C4 symmetry. **(H)** Select regions of the cryo-EM density map highlighting the T1 domain, CBD helix, presumed detergent densities observed in the channel, P-loop, S1-S6 helices, and the selectivity filter. Source data are provided as a Source Data file.

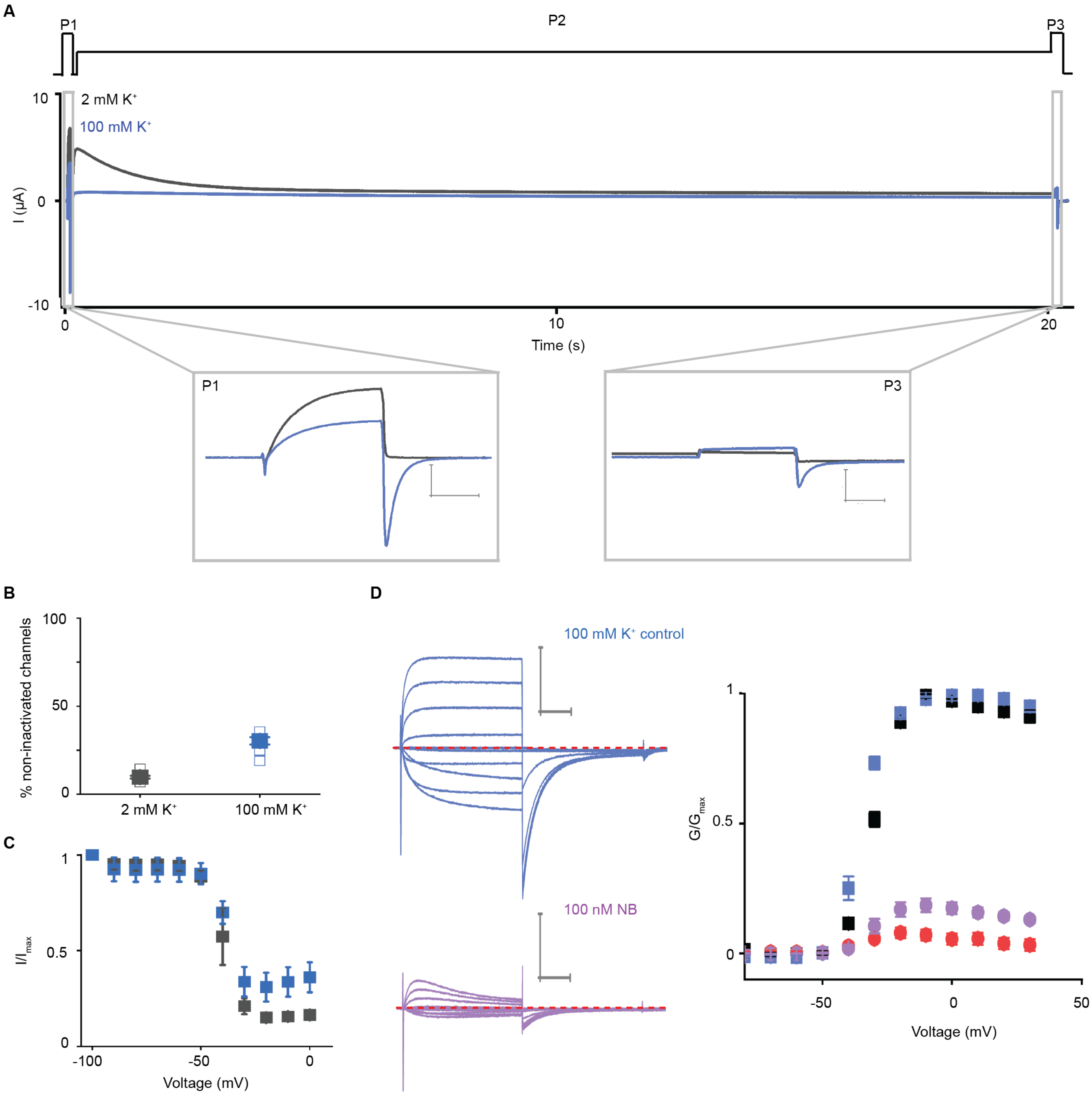

A
P1
P2
P3
10
2 mM K+
100 mM K+
I (μA)
0
-10
0
10
20
Time (s)
P1
P3
B
% non-inactivated channels
100
50
0
2 mM K+
100 mM K+
C
I/Imax
1
0.5
0
-100
-50
0
Voltage (mV)
D
100 mM K+ control
100 nM NB
G/Gmax
1
0.5
0
-50
0
50
Voltage (mV)

Supplementary Figure 2. Influence of high external K^+ on inactivation of Kv1.3 and inhibition by the nanobody.

(**A**) Kv1.3 current traces obtained using a three-pulse protocol (protocol insert) with varying external K^+ concentrations. The first pulse was to +30 mV (P1 inset), followed by a second pulse (P2) to 0 mV for 20 sec to allow channels to inactivate, and then a final third pulse (P3 inset) to +30 mV to assess the extent of inactivation. Holding voltage was -100 mV. Traces were obtained using P/-4 subtraction. (**B**) Percentage of non-inactivated currents at 0 mV in 2 mM external K^+ (black squares) and 100 mM external K^+ (blue squares) obtained from experiments like that shown in panel A. Percentage of non-inactivated currents was calculated by normalizing the steady-state current in P3 by the steady-state current value from the same trace in P1. Open symbols are individual experiments, filled symbols show averages (n=9) and error bars are SEM. (**C**) Steady-state inactivation relationships obtained in 2 mM external K^+ (black squares; n=3) and 100 mM external K^+ (blue squares; n=3) obtained by measuring the steady-state current in P3 (+30 mV) normalized to that in P1 (+30 mV). Measurements were made using a three-pulse protocol as in panel A but varying the voltage in P2 (-100 to 0 mV) without leak subtraction. Error bars are SEM. (**D**) Kv1.3 current traces obtained in 100 mM external K^+ from a family of depolarizing pulses ranging from -80 mV to +30 mV in control conditions (blue) and in the presence of 100 nM nanobody (NB) A0194009G09 (purple). Holding voltage was -80 mV and tail voltage was -50 mV. Traces were obtained using P/-4 subtraction. Red dotted line denotes zero current level. Scale bar indicates 2 μA and 50 ms. G-V relations obtained in control 100 mM external K^+ solutions (blue squares; n=4) or 100 nM NB (purple circles; n=4) were obtained by measuring the peak of the tail current at -50 mV and normalizing to the maximum tail current in control solution. G-V relations in 2 mM external K^+ control solutions (black squares) and with 100 nM NB (orange circles) are also shown as in Figure 3 for comparison purposes. Error bars are SEM. Source data are provided as a Source Data file.

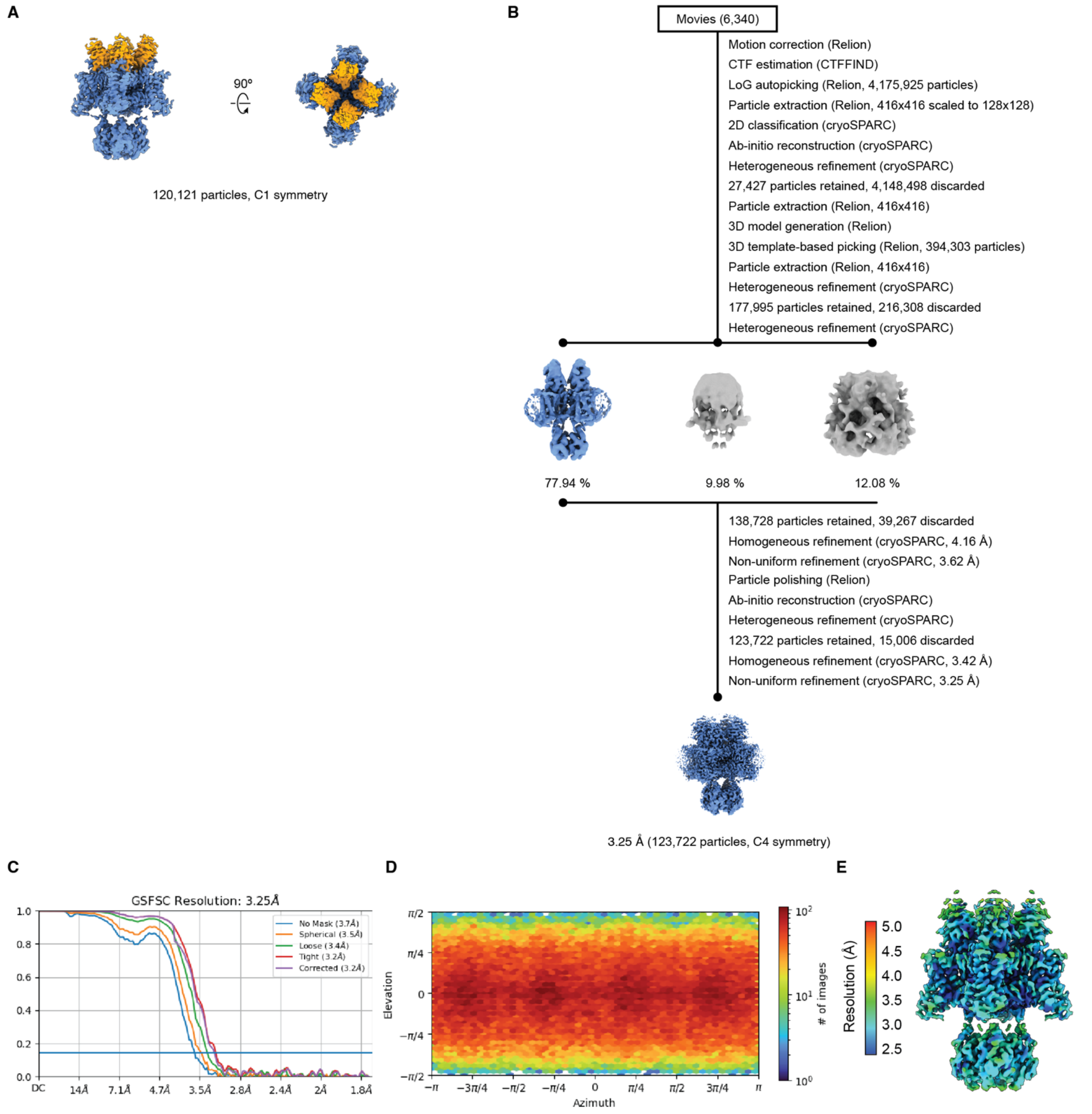

Supplementary Figure 3. Structure determination for human Kv1.3 with nanobody A0194009G09. (A) Cryo-EM density map of Kv1.3 with nanobody processed with C1 symmetry. The channel is colored in blue and the nanobodies in orange. **(B)** Data processing workflow for Kv1.3 in complex with A0194009G09 nanobodies. Cryo-EM density maps are color-coded as blue or gray according to whether they are retained or discarded, respectively. The percentage of particles in each class from heterogeneous refinement is given. **(C)** Fourier shell correlation (FSC) curves as generated by cryoSPARC. **(D)** Angular distribution plot for particles in the reconstruction as generated by cryoSPARC. **(E)** Local resolution heat map. Data was processed with C4 symmetry. Source data are provided as a Source Data file.

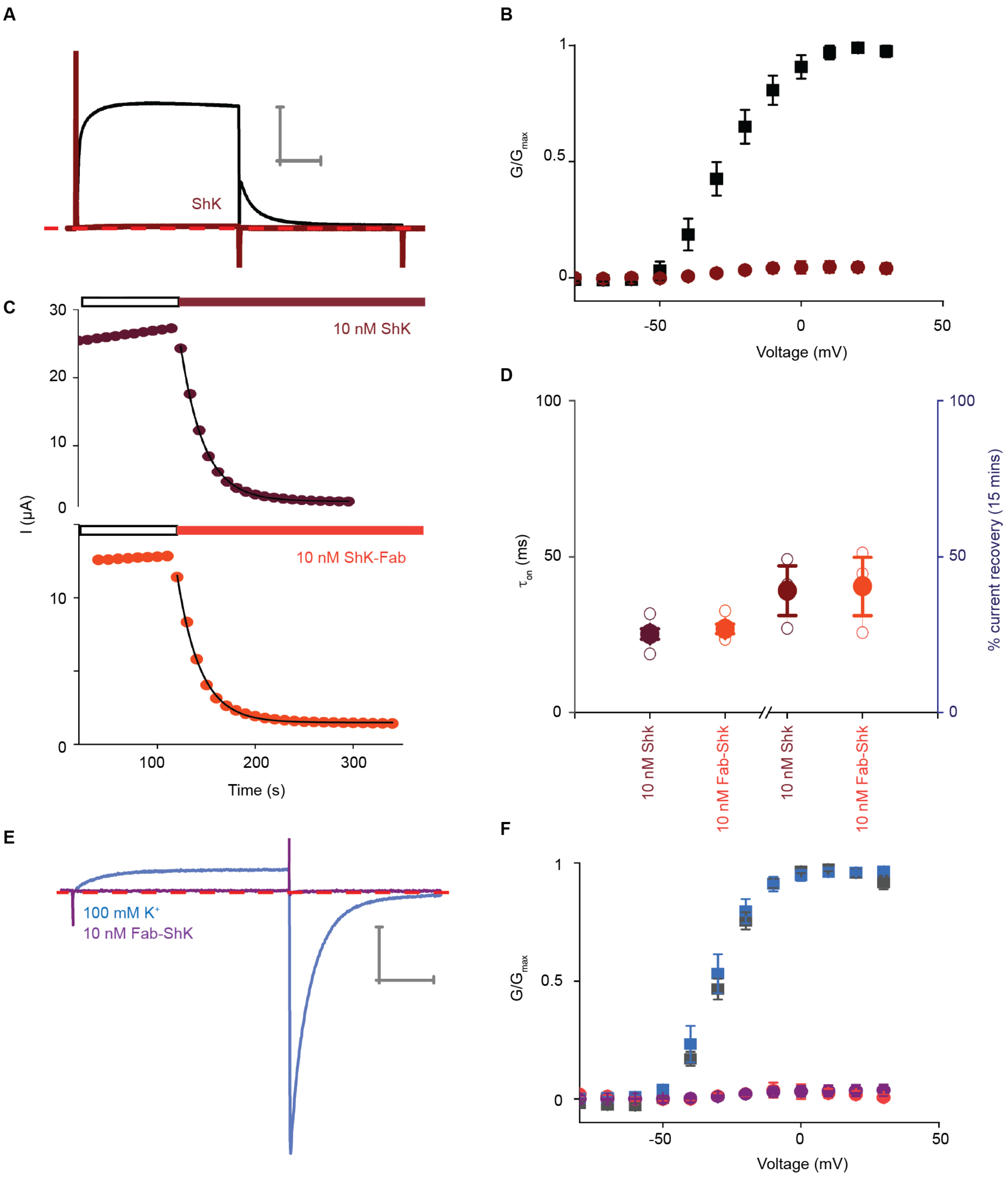

A
ShK
B
1
0.5
0
G/Gmax
-50
0
50
Voltage (mV)
C
30
20
10
0
10 nM ShK
I (μA)
10
0
10 nM ShK-Fab
100
200
300
Time (s)
D
100
50
0
τon (ms)
100
50
0
% current recovery (15 mins)
10 nM Shk
10 nM Fab-Shk
10 nM Shk
10 nM Fab-Shk
E
100 mM K+
10 nM Fab-ShK
F
1
0.5
0
G/Gmax
-50
0
50
Voltage (mV)

Supplementary Figure 4. Functional characterization of ShK and Fab-ShK.

(**A**) Kv1.3 current traces obtained by depolarizations to 0 mV in control 2 mM external K^+ solution (black) and in the presence of 10 nM ShK (maroon). Holding voltage was -80 mV and tail voltage was -50 mV. Traces were obtained using P/-4 subtraction. Red dotted lined denotes zero current level. Scale bar indicates 5 μA and 50 ms. (**B**) G-V relations obtained in control 2 mM external K^+ solution (black squares; n=3) or 10 nM ShK (maroon circles; n=3) by measuring the peak of the tail current at -50 mV and normalizing it to the maximum tail current in control solution. Error bars are SEM. (**C**) Time courses for onset of inhibition by ShK and Fab-ShK in 2 mM external K^+ solution as indicated. Peak currents were measured during 500 ms pulses to +40 mV from a holding potential of -80 mV. The perfusion of ShK or Fab-ShK at a concentration of 10 nM is indicated by the colored bar. Time scales in seconds were synchronized for plotting purposes. Solid black lines are fits to a single exponential function to the time course (see Methods). (**D**) Comparison of onset of inhibition (τ_{on}) for 10 nM ShK and Fab-ShK and current recovery after removing toxins from the external solution for 15 min. Mean τ_{on} values are shown in filled circles and individual measurements as open circles. τ_{on} values were obtained from single exponential fits to the time courses as shown in panel (C). In all instances, n = 3 and error bars are S.E.M. (**E**) Kv1.3 current traces obtained by depolarizations to 0 mV from control solution with 100 mM external K^+ (blue) and in the presence of 10 nM Fab-ShK (purple). Holding voltage was -80 mV and tail voltage was -50 mV. Traces were obtained using P/-4 subtraction. Red dotted lined denotes zero current level. Scale bar indicates 1 μA and 50 ms. (**F**) G-V relations obtained in control solution with 100 mM external K^+ (blue squares; n=4) and 10 nM Fab-ShK (purple circles; n=4) by measuring the peak of the tail current at -50 mV and normalizing it to the maximum tail current in control solution. G-V relations in 2 mM external K^+ (black squares) and with 10 nM Fab-ShK (orange circles) are also shown as in Figure 5 for comparison. Error bars are SEM. Source data are provided as a Source Data file.

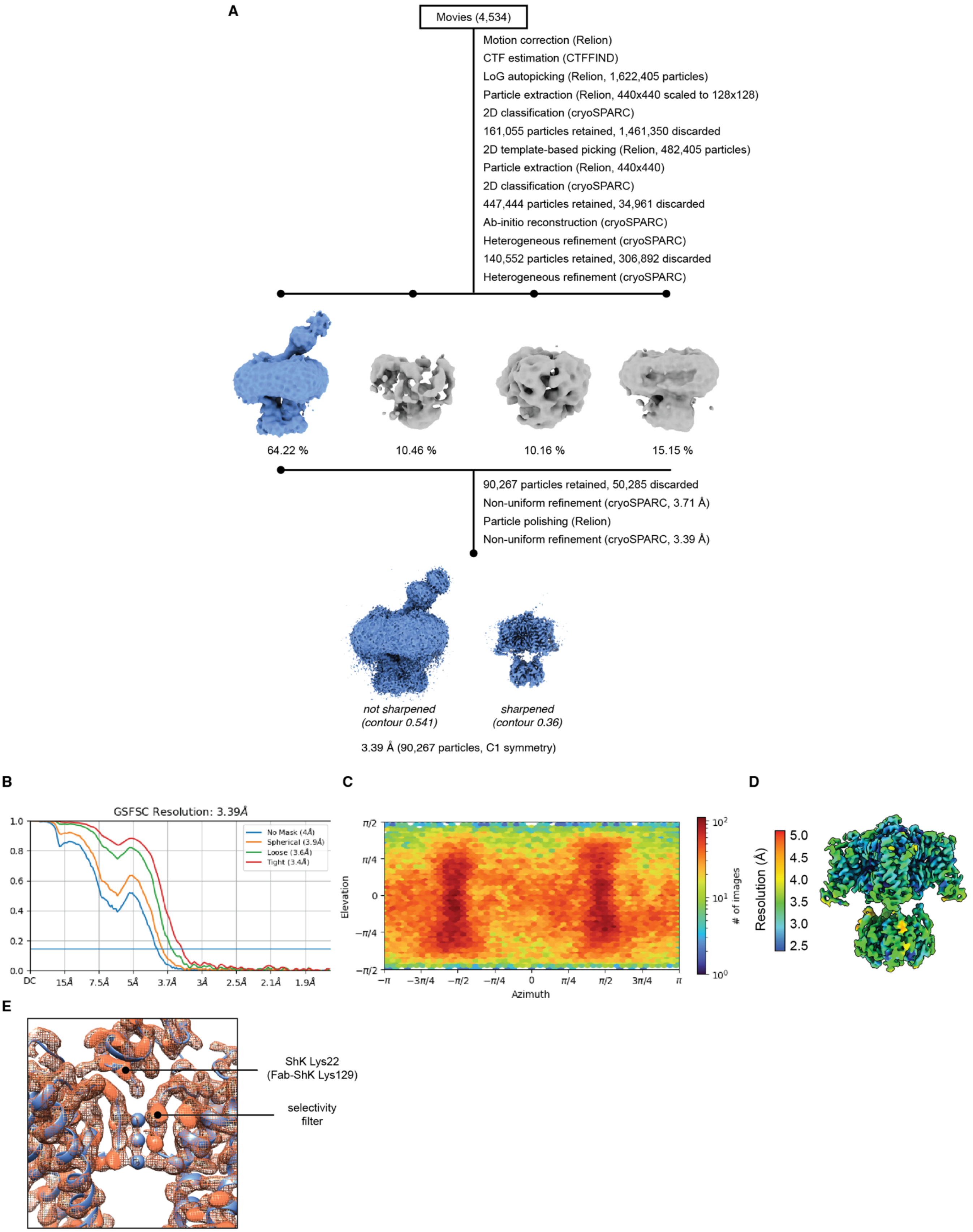
A
Movies (4,534)
Motion correction (Relion)
CTF estimation (CTFFIND)
LoG autopicking (Relion, 1,622,405 particles)
Particle extraction (Relion, 440x440 scaled to 128x128)
2D classification (cryoSPARC)
161,055 particles retained, 1,461,350 discarded
2D template-based picking (Relion, 482,405 particles)
Particle extraction (Relion, 440x440)
2D classification (cryoSPARC)
447,444 particles retained, 34,961 discarded
Ab-initio reconstruction (cryoSPARC)
Heterogeneous refinement (cryoSPARC)
140,552 particles retained, 306,892 discarded
Heterogeneous refinement (cryoSPARC)
64.22 %
10.46 %
10.16 %
15.15 %
90,267 particles retained, 50,285 discarded
Non-uniform refinement (cryoSPARC, 3.71 Å)
Particle polishing (Relion)
Non-uniform refinement (cryoSPARC, 3.39 Å)
not sharpened
(contour 0.541)
sharpened
(contour 0.36)
3.39 Å (90,267 particles, C1 symmetry)
B
GSFSC Resolution: 3.39Å
No Mask (4Å)
Spherical (3.9Å)
Loose (3.6Å)
Tight (3.4Å)
1.0
0.8
0.6
0.4
0.2
0.0
DC
15Å
7.5Å
5Å
3.7Å
3Å
2.5Å
2.1Å
1.9Å
C
Elevation
Azimuth
# of images
π/2
π/4
0
−π/4
−π/2
−π
−3π/4
−π/2
−π/4
0
π/4
π/2
3π/4
π
D
Resolution (Å)
5.0
4.5
4.0
3.5
3.0
2.5
E
ShK Lys22
(Fab-ShK Lys129)
selectivity
filter

Supplementary Figure 5. Structure determination for human Kv1.3 with Fab-ShK from the MNT-002 antibody.

(A) Data processing workflow for Kv1.3 in complex with Fab-ShK from the MNT-002 antibody. Cryo-EM density maps are color-coded as blue or gray according to whether they are retained or discarded, respectively. The percentage of particles in each class from heterogeneous refinement is given. The final map is shown without sharpening and at low contour to visualize the Fab density, and with sharpening and at high contour to visualize high-resolution structural features. **(B)** Fourier shell correlation (FSC) curves as generated by cryoSPARC. **(C)** Angular distribution plot for particles in the reconstruction as generated by cryoSPARC. **(D)** Local resolution heat map. Data was processed with C1 symmetry. **(E)** Kv1.3 map with model for the selectivity filter and ShK region of the structure. The structure highlights the potassium ions and key lysine residue of ShK that is coordinated by the selectivity filter. The lysine is position 22 in ShK or position 129 in the Fab-ShK fusion. The front and rear Kv1.3 subunits are omitted for clarity. Source data are provided as a Source Data file.

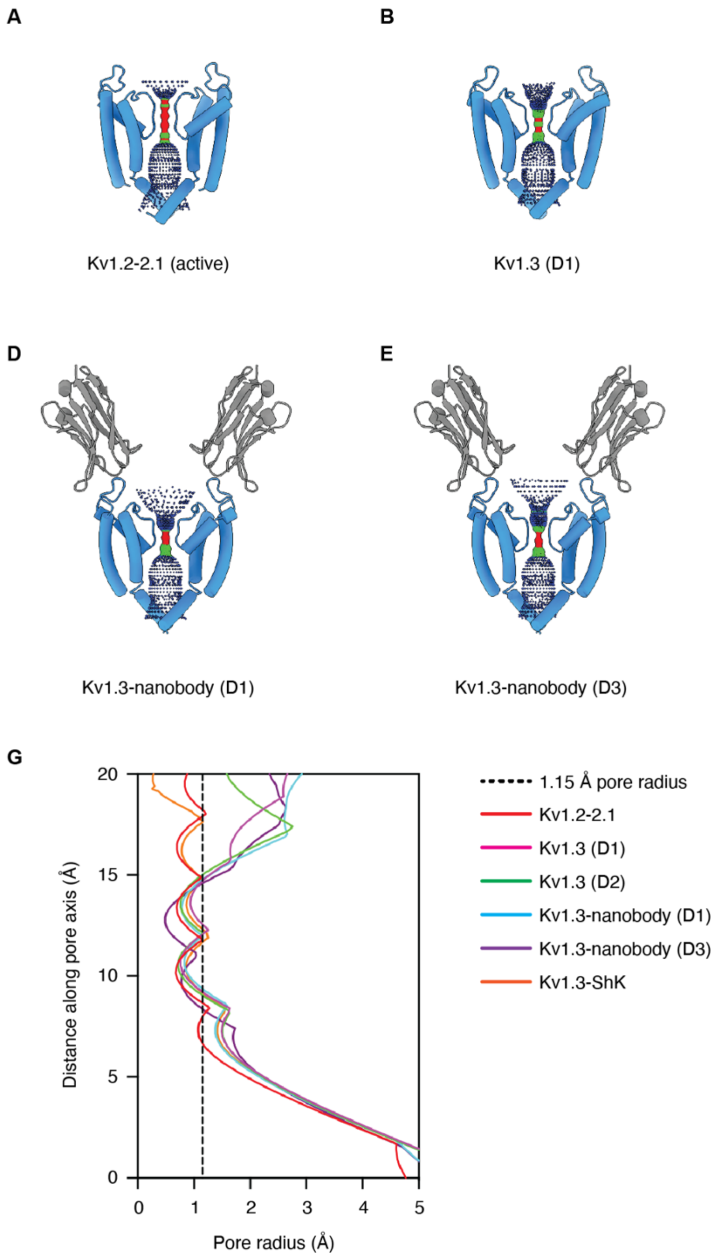

Supplementary Figure 6. Visualization of pore profiles.

(A-F) Visualizations of the pore structures for Kv1.2-2.1 (A), Kv1.3 in D1 conformation (B), Kv1.3 in D2 conformation (C), Kv1.3 with nanobodies in D1 conformation (D), Kv1.3 with nanobodies in D3 conformation (E), and Kv1.3 with ShK from Fab-ShK (F). Each structure is shown with only two subunits for visual clarity. Panels (D) and (E) show the Kv1.3 pore with two nanobodies, and panel (F) shows the Kv1.3 pore with the ShK toxin. **(G)** Plot of pore radius along the length of each pore structure in (A-F).

A

Comparison of Kv1.2-2.1 to Kv1.3 structures from the present study

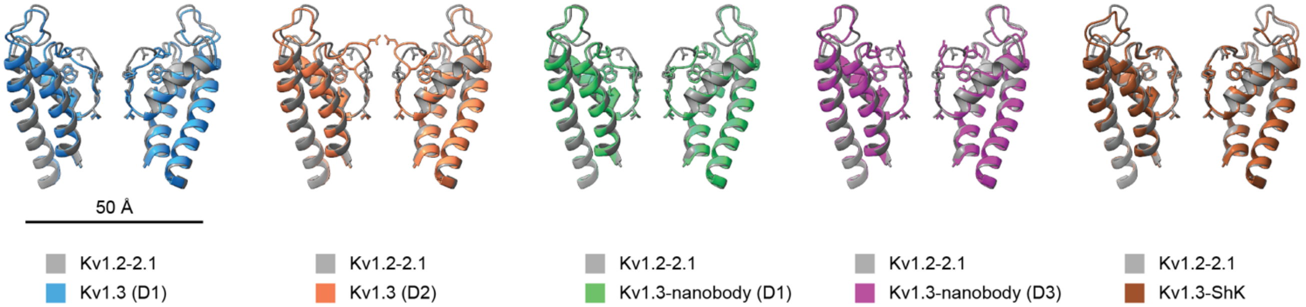

B

Comparison of Kv1.3 (D1) to other Kv1.3 dilated pore conformations

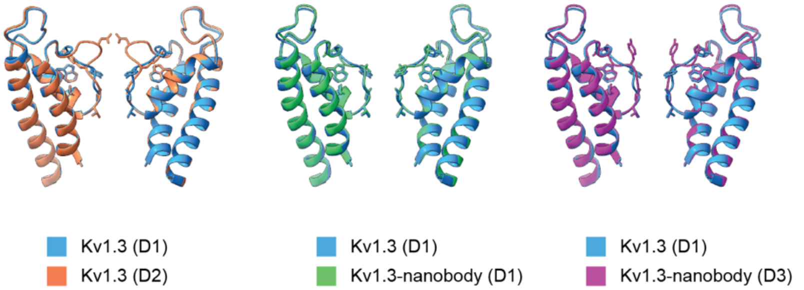

C

Comparison of Kv1.3 (D2) to other “D2-like” pore structures

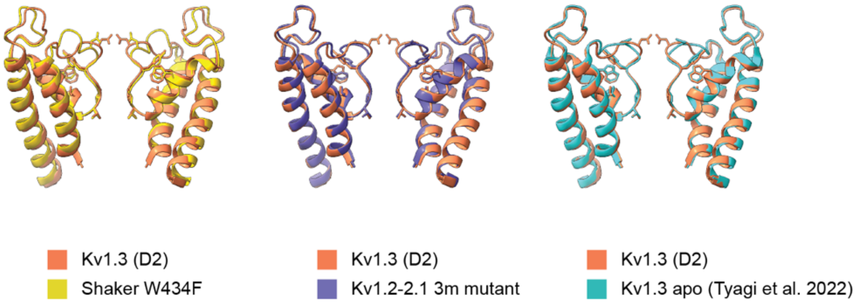

D

Comparison of Kv1.3-ShK to Kv1.3-dalazatide

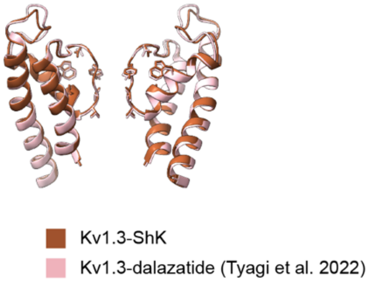

Supplementary Figure 7. Comparisons of pore conformations for Kv1.3, Kv1.2-2.1 and Shaker. **(A)** Structural alignments of pore regions for Kv1.2-2.1 (Long *et al.* 2007 *Nature*) and Kv1.3 structures (present study). **(B)** Structural alignments for Kv1.3 (D1) with Kv1.3-nanobody (D1 and D3). This comparison highlights the distinctions between D1, D2, and D3 loop conformations, and shows that the unbound and nanobody-bound structures both sample the D1 conformation. **(C)** Structural alignments for Kv1.3 (D2) with the Shaker W434F mutant (Tan *et al.* 2022 *Sci. Adv.*), the Kv1.2-2.1 3m mutant (Reddi *et al.* 2022 *Sci. Adv.*), and Kv1.3 apo (Tyagi *et al.* 2022 *Proc. Natl. Acad. Sci. USA*). **(D)** Structural alignments for Kv1.3-ShK (present study) with Kv1.3-dalazatide (Tyagi *et al.* 2022 *Proc. Natl. Acad. Sci. USA*). All of the structures are shown in cartoon style, but with the TVGYG motif shown with sticks in all structures. Sticks are also shown for Trp362 and Ser367 (Kv1.2-2.1), or Trp436 and Thr441 (Kv1.3), or Trp434 and Thr439 (Shaker), or Phe362 and Thr367 (Kv1.2-2.1 3m mutant).

	Kv1.3 D1 (EMDB-25416) (PDB 7SSX)	Kv1.3 D2 (EMDB-25416) (PDB 7SSY)	Kv1.3/NB D1 (EMDB-25417) (PDB 7SSZ)	Kv1.3/NB D3 (EMDB-25417) (PDB 8DFL)	Kv1.3/Fab-ShK (EMDB-25414) (PDB 7SSV)
**Data collection and processing**					
Magnification	105000	105000	105000	105000	105000
Voltage (kV)	300	300	300	300	300
Electron exposure (e–/Å^2)	53.7-54.23	53.7-54.23	50.93-55.97	50.93-55.97	51.36
Defocus range (μm)	1.3-2.0	1.3-2.0	1.3-2.0	1.3-2.0	1.3-2.0
Pixel size (Å)	0.426	0.426	0.426	0.426	0.426
Symmetry imposed	C4	C4	C4	C4	C1
Initial particle images (no.)	8385397	8385397	4175925	4175925	1622405
Final particle images (no.)	190793	190793	123722	123722	90267
Map resolution (Å)	2.89	2.89	3.25	3.25	3.39
FSC threshold	0.143	0.143	0.143	0.143	0.143
Map resolution range (Å)	2.89	2.89	3.25	3.25	3.39
**Refinement**					
Initial model used (PDB code)	2R9R	2R9R	2R9R, 6V80	2R9R, 6V80	2R9R, 6OO0
Model resolution (Å)					
FSC threshold					
Model resolution range (Å)					
Map sharpening *B* factor (Å^2)	124.4	124.4	124.7	124.7	90.4
Model composition					
Non-hydrogen atoms	11287	11287	15291	11287	14928
Protein residues	1384	1384	1908	1384	1874
Ligands	3	3	3	3	3
B factors (Å^2)					
Protein	29.67	29.67	117.51	117.51	76.69
Ligand	20.20	20.20	30.0	30.0	20.20
R.m.s. deviations					
Bond lengths (Å)	0.012	0.012	0.014	0.014	0.017
Bond angles (°)	1.522	1.485	1.811	1.770	1.803
Validation					
MolProbity score	1.81	1.48	2.38	2.35	2.31
Clashscore	3.61	3.04	7.11	6.45	6.16
Poor rotamers (%)	2.61	0.65	6.31	6.55	5.21
Ramachandran plot					
Favored (%)	95	94.41	94.46	94.46	93.43
Allowed (%)	5	5.59	5.54	5.54	6.57
Disallowed (%)	0	0	0	0	0

Supplementary Table 1. Cryo-EM data collection, refinement and validation statistics.

**Tetramers used for symmetry expansion**	190,793
**Subunits generated by symmetry expansion**	763,172
**Subunits classified as D1**	107,046
**Subunits classified as D2**	217,344
**Subunits in conformationally mixed or uninterpretable classes (discarded)**	438,782
**Tetramers with all 4 subunits identified**	6,598
**Tetramers with 1,2 or 3 subunits not identified (discarded)**	184,195
**D1:D2 ratios in tetramers with all 4 subunits identified:**	
0:4	1,752 tetramers (26.55%)
1:3	2,032 tetramers (30.8%)
2:2	1,618 tetramers (24.52%)
3:1	900 tetramers (13.64%)
4:0	296 tetramers (4.49%)
**D1 subunits analyzed**	9,152 (34.68%)
**D2 subunits analyzed**	17,240 (65.32%)

Supplementary Table 2. Conformational analysis of unbound Kv1.3.

Statistics from conformational analysis of unbound Kv1.3. The set of tetramer particles used as input for the analysis is the same as used for in the reported tetramer structure and contains 190,793 particles (**Supplementary Fig. 1**).
