# Supplementary material for: Structures of the T cell potassium channel Kv1.3 with immunoglobulin modulators
Source: Nat Commun. 2022 Jul 4;13:3854. doi: 10.1038/s41467-022-31285-5 (PMC9253088; doi:10.1038/s41467-022-31285-5)
Supplement: Supplementary file 3 — Reporting Summary [file 41467_2022_31285_MOESM3_ESM.pdf]

nature portfolio

Corresponding author(s): Joel Meyerson

Last updated by author(s): June 3, 2022

Reporting Summary

Nature Portfolio wishes to improve the reproducibility of the work that we publish. This form provides structure for consistency and transparency in reporting. For further information on Nature Portfolio policies, see our Editorial Policies and the Editorial Policy Checklist.

Statistics

For all statistical analyses, confirm that the following items are present in the figure legend, table legend, main text, or Methods section.

n/a	Confirmed	
☐	☒	The exact sample size (*n*) for each experimental group/condition, given as a discrete number and unit of measurement
☒	☐	A statement on whether measurements were taken from distinct samples or whether the same sample was measured repeatedly
☒	☐	The statistical test(s) used AND whether they are one- or two-sided<br>*Only common tests should be described solely by name; describe more complex techniques in the Methods section.*
☒	☐	A description of all covariates tested
☒	☐	A description of any assumptions or corrections, such as tests of normality and adjustment for multiple comparisons
☐	☒	A full description of the statistical parameters including central tendency (e.g. means) or other basic estimates (e.g. regression coefficient) AND variation (e.g. standard deviation) or associated estimates of uncertainty (e.g. confidence intervals)
☒	☐	For null hypothesis testing, the test statistic (e.g. *F*, *t*, *r*) with confidence intervals, effect sizes, degrees of freedom and *P* value noted<br>*Give P values as exact values whenever suitable.*
☒	☐	For Bayesian analysis, information on the choice of priors and Markov chain Monte Carlo settings
☒	☐	For hierarchical and complex designs, identification of the appropriate level for tests and full reporting of outcomes
☒	☐	Estimates of effect sizes (e.g. Cohen's *d*, Pearson's *r*), indicating how they were calculated

Our web collection on statistics for biologists contains articles on many of the points above.

Software and code

Policy information about availability of computer code

Data collection	Leginon, pCLAMP 10
Data analysis	Relion, cryoSPARC, Chimera, ChimeraX, Coot, Origin, CTFFIND4

For manuscripts utilizing custom algorithms or software that are central to the research but not yet described in published literature, software must be made available to editors and reviewers. We strongly encourage code deposition in a community repository (e.g. GitHub). See the Nature Portfolio guidelines for submitting code & software for further information.

Data

Policy information about availability of data

All manuscripts must include a data availability statement. This statement should provide the following information, where applicable:

- Accession codes, unique identifiers, or web links for publicly available datasets
- A description of any restrictions on data availability
- For clinical datasets or third party data, please ensure that the statement adheres to our policy

The cryo-EM density maps and models for Kv1.3, Kv1.3 with nanobody A0194009G09, and Kv1.3 with the Fab-ShK from MNT-002 have been deposited in the Protein Data Bank (PDB) and Electron Microscopy Data Bank and will be released upon publication. The PDB accession codes are 7SSX (Kv1.3 D1), 7SSY (Kv1.3 D2), 7SSZ (Kv1.3 with nanobody D1), 7ST0 (Kv1.3 with nanobody D3) and 7SSV (Kv1.3 with Fab-ShK) and the EMD accession codes are EMD-25416 (Kv1.3 map, D1 subunit map, D2 subunit map), EMD-25417 (Kv1.3 with nanobody) and EMD-25414 (Kv1.3 with Fab-ShK).

Cryo-EM maps, models and validation reports are also made available with this manuscript for download using this link:

https://wcm.box.com/s/epcoxn0pzizxdskplnb3henjoqyx05yn

Field-specific reporting

Please select the one below that is the best fit for your research. If you are not sure, read the appropriate sections before making your selection.

☒ Life sciences ☐ Behavioural & social sciences ☐ Ecological, evolutionary & environmental sciences

For a reference copy of the document with all sections, see nature.com/documents/nr-reporting-summary-flat.pdf

Life sciences study design

All studies must disclose on these points even when the disclosure is negative.

Sample size	Sample sizes were not predetermined for this study. Cryo-EM dataset sizes were determined by the need to attain target structural resolutions.
Data exclusions	The exclusions were pre-established and particles were removed if they resided in uninterpretable averages.
Replication	Protein purification, SDS-PAGE, and cryo-EM were performed independently several times with the same results. All attempts at replication were successful.
Randomization	Our experiments were not randomized. This statistical consideration is not relevant to our study because of the nature of biochemical, structural, and electrophysiological experiments performed in the work.
Blinding	The investigators were not blinded. Blinding is not technically or practically feasible for the experiments in this work.

Reporting for specific materials, systems and methods

We require information from authors about some types of materials, experimental systems and methods used in many studies. Here, indicate whether each material, system or method listed is relevant to your study. If you are not sure if a list item applies to your research, read the appropriate section before selecting a response.

Materials & experimental systems

n/a	Involved in the study	
☐	☒	Antibodies
☐	☒	Eukaryotic cell lines
☒	☐	Palaeontology and archaeology
☒	☐	Animals and other organisms
☒	☐	Human research participants
☒	☐	Clinical data
☒	☐	Dual use research of concern

Methods

n/a	Involved in the study	
☒	☐	ChIP-seq
☒	☐	Flow cytometry
☒	☐	MRI-based neuroimaging

Antibodies

Antibodies used	MNT-002 antibody Fab fragments were used. They were generated in the lab as described in the methods section.
Validation	Antibody binding was validated with electrophysiology and cryo-EM.

Eukaryotic cell lines

Policy information about cell lines

Cell line source(s)	ATCC CRL-3022
Authentication	Authenticated by manufacturer.
Mycoplasma contamination	Cells tested for mycoplasma by manufacturer.
Commonly misidentified lines (See ICLAC register)	None.
